# Palliative Care Team Involvement Is Associated with Changes in Nutrition Impact Symptoms and Eating-Related Distress in Patients with Advanced Cancer: A Multicenter Prospective Observational Study

**DOI:** 10.3390/nu18132174

**Published:** 2026-07-04

**Authors:** Koji Amano, Saori Koshimoto, Tatsuma Sakaguchi, Akihiro Tokoro, Sayaka Arakawa, Takashi Takeuchi, Naoharu Mori, Yoshinobu Matsuda, Eriko Satomi, Haruka Harano, Mitsunori Miyashita

**Affiliations:** 1 Department of Supportive and Palliative Care, Osaka International Cancer Institute, 3-1-69 Otemae, Chuo-ku, Osaka 541-8567, Japan; haruka.harano@oici.jp; 2 Liaison Psychiatry and Psycho-oncology Unit, Department of Psychiatry and Behavioral Sciences, Graduate School of Medical and Dental Sciences, Institute of Science Tokyo 1-5-45 Yushima, Bunkyo-ku, Tokyo 113-8510, Japan; skoshimoto-rd@umin.ac.jp (S.K.); okaspsyc@tmd.ac.jp (T.T.); 3 Faculty of Health Promotional Sciences, Department of Health and Nutritional Sciences, Tokoha University, 1230 Miyakoda-cho, Hamana-ku, Hamamatsu 431-2101, Shizuoka, Japan; 4 Department of Surgery, Kansai Medical University Kori Hospital, 8-45 Korihondori-cho, Neyagawa 572-8551, Osaka, Japan; sakaguct@kouri.kmu.ac.jp; 5 Department of Palliative and Supportive Medicine, Graduate School of Medicine, Aichi Medical University, 1-1 Yazakokarimata, Nagakute 480-1195, Aichi, Japan; nmori@aichi-med-u.ac.jp; 6 Department of Psychosomatic Internal Medicine and Supportive and Palliative Care Team, NHO Kinki Chuo Chest Medical Center, 1180 Nagasone-cho, Kita-ku, Sakai 591-8555, Osaka, Japan; tokoro.akihiro.qb@mail.hosp.go.jp (A.T.); matsuda.yoshinobu.tx@mail.hosp.go.jp (Y.M.); 7 Department of Palliative Medicine, National Cancer Center Hospital, 5-1-1 Tsukiji, Chuo-ku, Tokyo 104-0045, Japan; sarakawa@ncc.go.jp (S.A.); esatomi@ncc.go.jp (E.S.); 8 Department of Palliative Nursing, Health Sciences, Tohoku University Graduate School of Medicine, 2-1 Seiryo-machi, Aoba-ku, Sendai 980-8575, Miyagi, Japan; miya@tohoku.ac.jp

**Keywords:** cancer, cancer cachexia, palliative care, nutrition impact symptom, dietary intake, eating-related distress

## Abstract

Objectives: Evidence on the effects of palliative care team interventions on cachexia remains limited. We examined the effects on nutrition impact symptoms (NISs), dietary intake, and eating-related distress (ERD) among advanced cancer patients. Methods: This was a multicenter prospective observational study. Participants completed the baseline survey and the follow-up survey one week later. They evaluated NISs, dietary intake, and ERD. We compared the patient-reported outcomes between the two surveys. We categorized the participants having four or more NISs with a score ≥ 4 into the High-NIS group (otherwise, the Low-NIS group) using their baseline scores. Changes in the number of symptoms with a score ≥ 4, the dietary intake, and the ERD scores were calculated. Multiple regression analyses were performed to identify the factors influencing the improvements in dietary intake and ERD. Results: A total of 123 patients were included. In all patients, pain, fatigue, lack of appetite, sadness, and ERD significantly improved (*p* = 0.001, 0.023, 0.043, 0.037, <0.001). In the High-NIS group, the number of symptoms with a score ≥ 4 significantly decreased (*p* = 0.009), dietary intake scores significantly increased (*p* = 0.037), and ERD scores significantly decreased (*p* = 0.001). The number of symptoms with a score ≥ 4 at baseline (B = 1.08, *p* = 0.044, 95% confidence interval 0.03 to 2.14) was the only independent factor influencing the improvements in dietary intake. Conclusions: NISs and ERD improvements were associated with the interventions. Dietary intake improvement was associated with a high baseline symptom burden.

## 1. Introduction

A large number of patients with advanced cancer are affected by cancer cachexia, a disease-related wasting disorder characterized by anorexia, decreased dietary intake, unintentional weight loss, skeletal muscle loss, general fatigue, and poor quality of life (QOL) [1,2,3,4]. Continuous systemic inflammation and global metabolic reprogramming driven by dynamic tumor–host regulatory networks and host–tumor interactions are considered the primary underlying mechanisms of this wasting process [1,2,3,4]. Cancer cachexia is associated with various physical and psychological symptoms, leading to profound psychosocial distress for these patients [5,6,7,8,9,10,11,12]. Considering the coexistence of nutrition impact symptoms (NISs) and eating-related distress (ERD), this syndrome severely restricts dietary intake, compromises nutritional status, and further deteriorates patient QOL [5,6,7,8,9,10,11,12].

Evidence-based clinical practice guidelines emphasize the importance of multimodal interventions that act synergistically to palliate cachexia-associated symptoms and distress, to improve patient well-being [13,14,15,16]. Specifically, these guidelines emphasize the need to mitigate NISs, increase dietary intake, and alleviate ERD through multimodal approaches. However, recent systematic reviews using studies conducted in multiple countries (e.g., the USA, European countries, and Japan) indicate that robust evidence supporting the efficacy of these multimodal interventions remains insufficient [17,18]. Furthermore, no standardized treatments have been established to effectively improve patient-reported outcomes (PROs) such as NISs, ERD, dietary intake, and QOL [17,18]. Therefore, in clinical supportive and palliative care, multidisciplinary teams routinely collaborate to deliver holistic, multimodal care leveraging each member’s specialized expertise in Japan [11].

Over the past two decades in Japan, academic society-led palliative care educational programs have been held regularly nationwide, standardizing and maintaining the quality of interventions provided by palliative care teams in designated cancer care hospitals [19,20,21]. A typical multidisciplinary palliative care team comprises physicians, nurses, pharmacists, registered dietitians, psychologists, and medical social workers who provide comprehensive care (e.g., symptom control, oral care, psychological support, medication changes, nutritional counseling, and family counselling) to patients with advanced cancer and their families [19,20,21]. We recently reported that the perceived need for holistic, multimodal care among patients referred to palliative care was associated with their NISs, anxiety, and distress [22]. Building on these findings, the primary aim of the present study was to evaluate the effects of conventional palliative care team interventions on NISs, dietary intake, and ERD in patients with advanced cancer. Moreover, we secondarily explored the associations of the severity of the patients’ baseline NISs with improvements in dietary intake and ERD.

## 2. Methods

### 2.1. Study Design, Settings, and Participants

This multicenter, prospective observational study was conducted across palliative care teams working at six designated cancer care hospitals in Japan between November 2023 and June 2024, using a self-reported questionnaire. All consecutive hospitalized patients who met the eligibility criteria were enrolled. Participants were asked to complete the baseline survey at the time of enrollment, and those who responded were asked to complete the follow-up survey one week later, considering the potential for rapid clinical changes in patients receiving palliative care [23].

The following inclusion criteria were established: (1) adult patients aged 18 years or older, (2) patients diagnosed with advanced cancer or hematologic malignancies, (3) patients who were referred to palliative care for the first time, (4) patients who were aware of their cancer diagnosis, and (5) patients who could complete a self-reported questionnaire written in Japanese. The following exclusion criteria were established: (1) patients who were forbidden to eat and drink by the attending physician for medical reasons, (2) patients who were too distressed to complete the questionnaire (as determined through an interview with the palliative care physician), or (3) patients who declined to participate.

### 2.2. Ethics Approval and Consent to Participate

This study adhered to the ethical standards outlined in the Declaration of Helsinki and the Ministry of Health, Labour and Welfare’s “Ethical Guidelines for Medical and Health Research Involving Human Subjects” [24]. Approval was obtained from the Institutional Review Board of Osaka University Hospital for all participating institutions (approval number 23226, approval date 29 September 2023). Patient participation required the completion and return of both the questionnaire and a consent form. Patients who declined to participate were asked to return the questionnaire marked “Declined to participate.”

### 2.3. Interventions and Measurements

The palliative care team interventions were not delivered according to a single study protocol across the six participating designated cancer care hospitals. However, the interventions in the participating hospitals were standardized by the academic society-led palliative care educational programs and clinical practice guidelines [19,20,21]. Symptom control, analgesic adjustment, antiemetic or laxative treatment, oral care, nutritional counselling, dietitian consultation, psychosocial support, family counselling, and medication review were routinely provided in each hospital.

Patient characteristics, including primary cancer sites, treatment status, Eastern Cooperative Oncology Group (ECOG) performance status [25], and other variables associated with cancer cachexia, were collected. Participants who did not undergo anticancer treatments or discontinued chemotherapy were categorized as “never treated/previous treatment.” The latest laboratory test results were obtained within one week before enrollment. Moreover, anthropometric data—height [m], body weight at enrollment [kg], and body weight 6 months prior to enrollment [kg]—were collected. The body mass index (BMI) was calculated by dividing the body weight [kg] by the height [m] squared. The weight loss rate (WLR) within the previous 6 months was calculated using the following formula: (body weight 6 months prior to enrollment [kg] − body weight at enrollment [kg])/body weight 6 months prior to enrollment [kg]) × 100. Patients with WLR > 5% or BMI < 20 kg/m^2^ + WLR > 2% were diagnosed with cachexia or refractory cachexia, following international diagnostic criteria [1].

The patients evaluated 19 NISs using an 11-point numerical rating scale (0, none; 1–3, mild; 4–6, moderate; 7–9, severe; 10, unbearable). Because there is no international consensus definition for NISs, making it unclear which specific symptoms qualify, an international multi-provider survey aimed at unifying the terminology advocated a tentative definition: NISs are symptoms that compromise patients’ desire or ability to eat, interfering with nutritional needs and increasing the risk of malnutrition, lean body mass loss, and impaired QOL [26]; the 19 items used in the present study were selected from the symptoms proposed by the international survey. Furthermore, the selected NISs have been validated and are supported in the clinical recommendations edited by the American Society for Parenteral and Enteral Nutrition as PRO measures [27].

The patients also reported their current dietary intake using the Ingesta-Verbal/Visual Analogue Scale (IVVAS) with a range of 0–10 (0, I eat nothing; 5, I eat half the usual amount; 10, I eat as usual). An IVVAS score ≤ 7 indicates that the patient has a nutritional risk for weight loss [28,29]. Having four or more NISs with a score ≥ 4 has been significantly associated with decreased dietary intake (as assessed using the IVVAS), depression (as diagnosed using the Patient Health Questionnaire-9) [30,31], and the perceived need for holistic, multimodal care in similar populations [22,32,33].

Additionally, the patients assessed their ERD using the Questionnaire for Eating-Related Distress among Patients with advanced cancer (QERD-P) [34]. The long-form QERD-P contains 21 items across 7 factors (3 items per factor), and each item is rated on a 7-point Likert scale; higher scores indicate worse distress. The short-form QERD-P only considers 7 items based on the 7 factors. The 7 factors are (1) reduced dietary intake, (2) reasons why I cannot eat, (3) becoming weaker, (4) insufficient information, (5) arguments with my family, (6) changes in appearance, and (7) time with my family. Having four or more NISs with a score ≥ 4 has also been significantly correlated with ERD (as assessed using the QERD-P) in this population [32].

Finally, patients’ QOL was evaluated using the European Organization for Research and Treatment of Cancer Core Quality of Life Questionnaire (EORTC QLQ-C30) [35].

### 2.4. Statistical Analysis

The sample size needed for a before-and-after comparison using a one-group paired *t*-test was calculated with an α error of 0.05, a statistical power of 0.8, and an effect size of 0.3. This yielded an adequate sample size of 90 cases. We aimed to enroll more than 180 patients, assuming a 50% attrition rate.

We compared the variables obtained in the baseline survey with those collected in the one-week follow-up survey using the Wilcoxon signed-rank test or McNemar’s test, where appropriate, to investigate the effects of palliative care team interventions on the improvements in NISs, dietary intake, ERD, and QOL.

Based on the baseline scores, we divided the patients into a High-NIS group (having four or more NISs with a score ≥ 4) and a Low-NIS group (having fewer than four NISs with a score ≥ 4). Then, we analyzed the inter-group differences regarding changes in the number of NISs with a score ≥ 4 and the improvement rates of each NIS using the Mann–Whitney U test or chi-squared test, where appropriate. We defined the improvement rate for an individual symptom as the percentage of patients who improved to a symptom score < 7 among those who had a score ≥ 7 at baseline and patients who improved to a score < 4 among those who had a score ≥ 4 at baseline. Moreover, we compared the changes and improvement rates for the dietary intake scores between the two groups in the same manner. We defined two types of dietary intake improvement rates: the percentage of patients shifting from an IVVAS score ≤ 7 to >7, and the percentage of individuals whose IVVAS score increased by 2 points. Finally, we calculated the within-group changes from baseline to follow-up for the number of symptoms with a score ≥ 4, the IVVAS scores, and the QERD-P scores using the Wilcoxon signed-rank test.

Multiple regression analysis was performed using the forced entry method to identify the factors influencing the improvement in dietary intake. The number of symptoms with a score ≥ 4 (0–3 vs. 4 or more), age, sex (male vs. female), primary cancer site (lung, gastrointestinal tract, vs. other), ECOG performance status (0/1, 2, vs. 3/4), and treatment status (pre-chemotherapy, chemotherapy, vs. never treated/previous treatment) were included in the multivariate analysis. Similarly, multiple regression analysis was performed to identify the factors influencing the improvement in ERD. These variables were tested in the multiple regression analysis because they were associated with dietary intake and ERD in similar populations and considered clinically important [32].

All analyses were calculated using SPSS software Version 28.0.1 (IBM, Tokyo, Japan). Results were considered significant if the *p*-value was <0.05.

## 3. Results

### 3.1. Patient Flow Diagram

**Figure 1:**
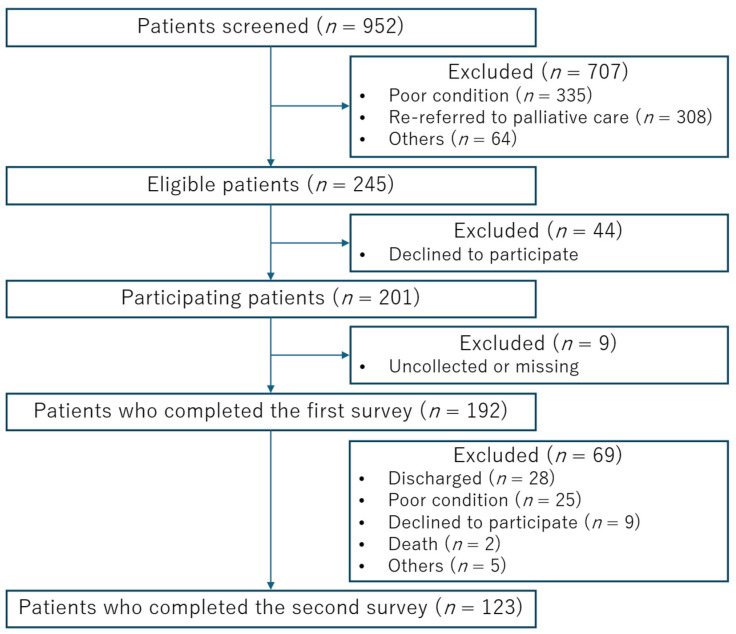
Flow diagram of the patient selection process in this study.

Of the 952 patients who were screened, 245 met the eligibility criteria. After 44 patients declined to participate, 201 patients were enrolled in the study, and 192 completed the baseline survey. Among them, 69 patients were excluded from the follow-up survey due to hospital discharge, clinical deterioration, death, or declined participation. Ultimately, 123 patients were included in the final analysis (Figure 1).

### 3.2. Patient Characteristics

Patients’ characteristics are shown in Table 1. The median age was 65.0 years. Females and males accounted for 55.3% and 44.7% of the cohort, respectively. The three most common primary cancer sites were the lung (21.1%), the liver, biliary system, and pancreas (15.4%), and the upper and lower gastrointestinal tracts (14.6%). The proportions of ECOG performance status 0 or 1, 2, 3, and 4 were 24.4%, 35.0%, 37.4%, and 3.3%, respectively. Notably, 73.2% of the patients were undergoing chemotherapy, and 27.6% of them were affected by cancer cachexia or refractory cachexia.

### 3.3. Baseline-to-Follow-Up Changes

A comparison of the symptom severity and dietary intake before and after palliative care team intervention across all patients is summarized in Table 2. Pain, fatigue, lack of appetite, and sadness improved significantly (*p* = 0.001, 0.023, 0.043, and 0.037, respectively), whereas oral pain worsened significantly (*p* = 0.039). However, no significant differences were observed in the number of symptoms with a score ≥ 4 or in the IVVAS scores.

A comparison of ERD before and after palliative care team intervention across all patients is shown in Table 3. All items included in the four factors associated with ERD (i.e., reduced dietary intake, reasons why I cannot eat, becoming weaker, insufficient information) improved significantly. Notably, total scores for both the long- and short-form QERD-P significantly decreased (both *p* < 0.001).

A comparison of QOL before and after palliative care team intervention across all patients is reported in Table 4. One patient was excluded from this analysis due to missing variables. Role functioning and global health status improved significantly (*p* = 0.036 and <0.001, respectively). Furthermore, fatigue, pain, sleep disturbance, and appetite loss also improved significantly (*p* = 0.001, <0.001, 0.027, and 0.023, respectively).

### 3.4. High-NIS Versus Low-NIS Comparisons

A comparison of the intervention effects between the High- and Low-NIS groups is summarized in Table 5. Significant differences were observed between the two groups regarding changes in both the number of symptoms with a score ≥ 4 and the overall number of improved symptoms (both *p* < 0.001). However, no significant differences were found for changes in IVVAS scores, dietary intake improvement rates, or QERD-P scores.

**Figure 2:**
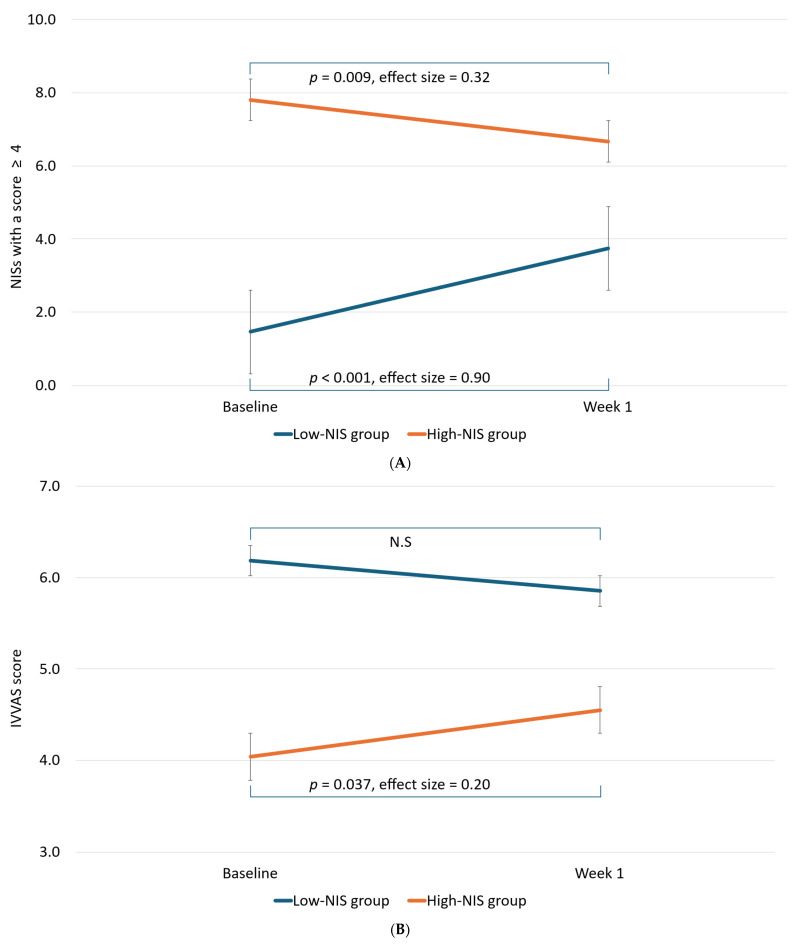

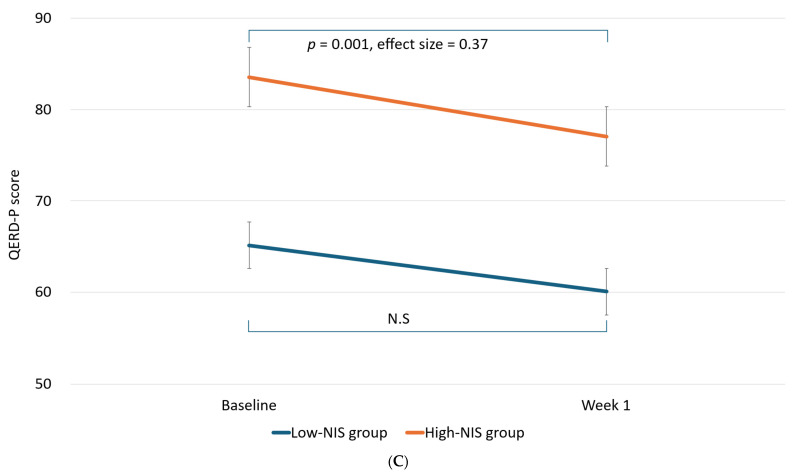
(**A**). Changes in the number of symptoms with a score ≥ 4 from baseline to follow-up in the High- and Low-NIS groups. The number of symptoms with a score ≥ 4 in the High-NIS group significantly decreased (*p* = 0.009), whereas that in the Low-NIS group significantly increased (*p* < 0.001). *p*-values refer to within-group comparisons. The error bars represent the standard error. NIS, nutrition impact symptom. (**B**). Changes in the IVVAS score from baseline to follow-up in the High- and Low-NIS groups. IVVAS scores significantly increased in the High-NIS group (*p* = 0.037). However, there was no significant change in the Low-NIS group. *p*-values refer to within-group comparisons. The error bars represent the standard error. IVVAS, Ingesta-Verbal/Visual Analogue Scale; NIS, nutrition impact symptom; N.S, not significant. (**C**). Changes in the QERD-P score from baseline to follow-up in the High- and Low-NIS groups. QERD-P scores significantly decreased in the High-NIS group (*p* = 0.001). However, there was no significant change in the Low-NIS group. *p*-values refer to within-group comparisons. The error bars represent the standard error. QERD-P, Questionnaire for Eating-Related Distress among Patients with advanced cancer; NIS, nutrition impact symptom; N.S, not significant.

Changes in the number of symptoms with a score ≥ 4, the IVVAS scores, and the QERD-P scores from the baseline to the follow-up in the High- and Low-NIS groups are shown in Figure 2A, Figure 2B, and Figure 2C, respectively. The number of symptoms with a score ≥ 4 significantly decreased in the High-NIS group (*p* = 0.009) but significantly increased in the Low-NIS group (*p* < 0.001). Moreover, the IVVAS scores significantly increased while the QERD-P scores significantly decreased in the High-NIS group (*p* = 0.037 and *p* = 0.001, respectively), whereas no significant changes were observed for these scores in the Low-NIS group.

### 3.5. Multiple Regression Analyses

Multiple regression analyses were performed to identify independent predictors of improved dietary intake and improved ERD (Table 6). The number of symptoms with a score ≥ 4 at baseline (B = 1.08, *p* = 0.044, 95% confidence interval [CI] 0.03 to 2.14) was the only factor that significantly influenced dietary intake improvement, and none of the factors were found to significantly influence ERD improvement.

## 4. Discussion

This study represents the first multi-institutional survey to examine the associations between conventional palliative care team interventions and improvements in NISs, dietary intake, and ERD among patients with advanced cancer. Furthermore, we investigated short-term changes after palliative care team involvement in dietary intake and ERD according to the baseline severity of the patients’ NISs.

Our results suggest that standard palliative care team interventions in Japan had beneficial effects on the management of several symptoms, including pain, fatigue, lack of appetite, sadness, and sleep disturbances, which are strongly associated with cancer cachexia [9,10,11,12]. Notably, the interventions seemed to effectively alleviate ERD, despite showing no significant impact on dietary intake. However, since no adjustment for multiple comparisons was applied in NISs, dietary intake, QERD-P items, and EORTC QLQ-C30 domains, the analyses of them were exploratory and nominal *p*-values should be interpreted cautiously.

The use of PROs to capture patients’ symptom burden and distress was a major strength of this evaluation. Structurally, a hospital-based palliative care team is a multidisciplinary unit equipped with specialized knowledge and skills to alleviate symptoms and optimize QOL for patients and their family members through targeted clinical consultations [36]. Systematic reviews have reported that palliative care teams are common worldwide and effective at managing pain, anxiety, and other symptoms, leading to reduced hospitalizations and healthcare costs [36,37], but few studies have used validated PRO measures to evaluate patients’ symptoms. A recent multicenter, prospective observational study conducted in Japan to explore the effectiveness of the hospital-based palliative care teams using PROs demonstrated significant clinical improvements (one week post-intervention) in pain, shortness of breath, lack of information, practical problems, and poor well-being [23]. The findings of the present study are consistent with those of the previous research and further address the effects of palliative care team interventions on NISs and ERD.

The results also show that having four or more NISs with a score ≥ 4 at baseline was significantly associated with post-intervention improvements in dietary intake. However, the High-NIS/Low-NIS comparison should be interpreted cautiously. Because these groups were defined by baseline symptom burden, the observed improvement in the High-NIS group might partly reflect regression to the mean. Although high baseline NIS burden was associated with larger observed short-term changes in dietary intake, the efficacy of the interventions should be further evaluated in baseline-adjusted or controlled studies in the near future.

Our previous study similarly revealed that the perceived need for multimodal care among advanced cancer patients referred to palliative care was associated with having four or more NISs with a score ≥ 4, anxiety, alongside heightened distress screened using the Hospital Anxiety and Depression Scale (HADS) and Distress/Impact Thermometers [22,38,39,40]. Collectively, these findings suggest that a threshold of four or more severe NISs serves as a reliable clinical indicator for initiating holistic, multimodal palliative care team interventions. Nonetheless, there remains an urgent need to develop and validate novel screening tools to identify patients with advanced cancer who are most likely to benefit from specialized palliative care, ensuring that evidence-based, multimodal frameworks are implemented in a more timely manner.

Several strengths and limitations of this study warrant consideration. The multicenter setting, the focus on a vulnerable palliative care population, and the use of PROs were strengths. Approximately 82% of the eligible candidates were enrolled, with a 96% completion rate for the baseline survey and a 61% retention rate at the one-week follow-up. Considering the inherent vulnerability of this cohort, these rates were relatively high. However, patients who died or were discharged alive within a week might have different symptom trajectories. Moreover, only 123 patients were included in the final analysis despite 952 screened patients. This might be one of the potential sources of bias. Since a patient with advanced cancer usually receives interventions provided by a palliative care team multiple times in Japan, we knew that limiting the eligibility criteria to patients receiving the first intervention would result in a big exclusion number. However, we believed that including patients who had already received palliative care interventions and those who had never received any palliative care interventions together would introduce a bigger bias, so we limited the study population to patients who were referred to palliative care for the first time. Additionally, because approximately 73% of the participants were concurrently receiving chemotherapy, our findings reflect real-world data from advanced cancer patients actively managed in designated cancer care hospitals across Japan. However, certain limitations restrict generalizability. The multicenter design of this study might introduce heterogeneity across institutions and palliative care teams. Moreover, this study was conducted in a single East Asian nation and was restricted to hospitalized patients. Furthermore, although palliative care team interventions are standardized in Japan, delivery frameworks and clinical protocols vary globally, and cultural or ethnic differences regarding distress must be acknowledged. Nevertheless, because pain and symptom management are fundamental to palliative care [36,37], the insights from this study hold international relevance. Furthermore, although this study was framed around cancer cachexia, only a subset of patients fulfilled cachexia/refractory cachexia criteria. As the current diagnosis of cachexia is based primarily on the weight loss rate, in patients with significant fluid retention (i.e., edema, ascites, pleural effusion), the weight loss rate might be underestimated [41], resulting in a failure to diagnose cachexia. A greater number of patients might have been in a cachectic state. Further research is necessary to validate the present findings and clarify their application to clinical oncology practices worldwide.

## 5. Conclusions

This study suggested that conventional palliative care team interventions significantly improved NISs and ERD, as well as the global health status, in patients with advanced cancer. Dietary intake was not significantly improved in the overall cohort; however, improvements were observed after palliative care team involvement among patients presenting with four or more severe NISs. Overall, these findings suggest that conventional palliative care team interventions provide a valuable framework for the supportive management of advanced cancer.

## Figures and Tables

**Table 1:** Patient characteristics (*n* = 123).

Age (years)	65.0 (53.0, 74.0)
Sex	
Female	68 (55.3)
Male	55 (44.7)
Primary cancer site	
Lung	26 (21.1)
Liver, biliary system, and pancreas	19 (15.4)
Upper and lower gastrointestinal tracts	18 (14.6)
Gynecologic	17 (13.8)
Breast	9 (7.3)
Urological	8 (6.5)
Head and neck	7 (5.7)
Hematological	6 (4.9)
Others	13 (10.6)
Treatment status	
Pre-chemotherapy	17 (13.8)
Chemotherapy	90 (73.2)
Never treated/previous treatment	16 (13.0)
ECOG performance status	
0 or 1	30 (24.4)
2	43 (35.0)
3	46 (37.4)
4	4 (3.3)
Body mass index (kg/m^2^)	20.8 (18.7, 24.3)
Weight loss rate over 6 months (%)	6.7 (0.8, 12.1)
Cachexia/refractory cachexia, yes	34 (27.6)
Pleural effusion, ascites, or edema affecting weight, yes	22 (17.9)
Albumin (g/dL)	3.2 (2.8, 3.7)
C-reactive protein (mg/dL)	2.6 (0.4, 7.3)

Values represent n (%) or the median (interquartile range). ECOG, Eastern Cooperative Oncology Group.

**Table 2:** Comparison of the symptom severity and dietary intake before and after palliative care team interventions (*n* = 123).

	Baseline	Week 1	*p*-Value	Effect Size
NISs				
Number of NISs with a score ≥ 4	6.0 ± 3.8	5.8 ± 4.0	0.817	0.04
Symptom severity score				
Oral pain	0.9 ± 1.9	1.2 ± 2.2	0.039	0.18
Pain	3.8 ± 3.4	2.8 ± 2.9	0.001	0.33
Shortness of breath	2.5 ± 2.7	2.1 ± 2.6	0.072	0.14
Fatigue	4.1 ± 2.9	3.6 ± 2.7	0.023	0.18
Drowsiness	3.6 ± 2.7	3.9 ± 2.7	0.213	0.11
Lack of appetite	4.9 ± 3.2	4.3 ± 3.0	0.043	0.19
Early satiety	5.1 ± 3.1	4.7 ± 3.1	0.204	0.11
Nausea	2.3 ± 2.9	1.8 ± 2.5	0.102	0.18
Vomiting	1.2 ± 2.5	0.9 ± 2.0	0.059	0.19
Constipation	3.7 ± 3.4	4.0 ± 3.4	0.393	0.08
Diarrhea	1.8 ± 2.7	1.6 ± 2.4	0.563	0.08
Abnormal taste	2.2 ± 3.1	2.2 ± 3.0	0.890	0.02
Abnormal smell	1.2 ± 2.2	1.5 ± 2.5	0.058	0.14
Dry mouth	2.9 ± 3.2	3.1 ± 3.2	0.226	0.08
Dental problems	1.7 ± 2.6	1.9 ± 2.9	0.492	0.04
Difficulty swallowing	1.4 ± 2.4	1.6 ± 2.5	0.293	0.07
Food bolus obstruction	1.8 ± 2.9	1.7 ± 2.6	0.838	0.02
Anxiety	3.5 ± 3.1	3.2 ± 2.8	0.237	0.11
Sadness	3.6 ± 2.8	3.1 ± 2.7	0.037	0.17
Dietary intake				
Dietary intake score	4.7 ± 2.7	4.9 ± 2.6	0.157	0.12
Proportion of patients with a dietary intake score ≤ 7	105 (85.4)	97 (78.9)	0.115	0.17

Values represent n (%) or the mean ± standard deviation. NISs were rated between 0 and 10. High scores indicate worse symptoms. Dietary intake was assessed using the IVVAS (10-point scale). High scores indicate better dietary intake. NIS, nutrition impact symptom; IVVAS, Ingesta-Verbal/Visual Analogue Scale.

**Table 3:** Comparison of the ERD before and after palliative care team interventions (*n* = 123).

	Baseline	Week 1	*p*-Value	Effect Size
**It is distressing that I cannot eat even though I want to eat more.**	4.6 ± 2.0	3.8 ± 2.1	<0.001	0.40
It is distressing that I cannot enjoy eating.	4.9 ± 1.9	4.1 ± 2.1	<0.001	0.40
It is distressing that I get full quickly and cannot eat enough.	4.4 ± 1.9	3.8 ± 2.0	<0.001	0.31
**I do not understand the reason why I cannot eat.**	3.3 ± 1.8	2.9 ± 1.8	0.011	0.25
I do not understand the reason why I do not have an appetite.	3.1 ± 1.7	2.9 ± 1.8	0.042	0.18
I do not understand the reason why I cannot eat enough.	3.1 ± 1.8	2.8 ± 1.7	0.045	0.17
**I am concerned that I will become weaker if I cannot eat.**	5.0 ± 1.9	4.3 ± 1.9	<0.001	0.32
I am concerned that I will lose muscle strength if I cannot eat.	5.2 ± 1.8	4.6 ± 1.9	<0.001	0.32
I am concerned that I will lose weight if I cannot eat.	4.8 ± 1.9	4.3 ± 1.9	0.007	0.27
**I have insufficient information about which nutrients I should prioritize.**	4.2 ± 1.8	3.8 ± 1.7	0.002	0.26
I have insufficient information about which nutrients I should avoid.	4.2 ± 1.7	3.7 ± 1.7	0.001	0.28
I have insufficient information about which nutritional supplements I should take.	4.0± 1.7	3.7 ± 1.7	0.031	0.17
**I have arguments with my family about food.**	2.5 ± 1.7	2.3 ± 1.7	0.256	0.08
I am troubled that my family seems to try to force me to eat.	2.3 ± 1.7	2.2 ± 1.6	0.519	0.05
I get frustrated with my family over food.	2.3 ± 1.6	2.2 ± 1.6	0.347	0.07
**It is hard for me that my appearance has changed a lot from before as I became thin.**	3.4 ± 2.0	3.2 ± 1.8	0.492	0.06
It is hard for me to be seen by others as so skinny.	3.2 ± 1.9	3.0 ± 1.7	0.218	0.10
It is hard to see myself as so skinny.	3.4 ± 1.9	3.2 ± 1.8	0.237	0.11
**I spend less time talking with my family because I do not eat with them.**	3.3 ± 1.9	3.4 ± 2.1	0.798	0.03
I spend less time enjoying with my family during meals.	3.8 ± 2.1	4.2 ± 2.0	0.065	0.18
I spend less time in daily life with my family because I cannot eat.	3.2 ± 2.0	3.8 ± 2.0	0.006	0.26
Total score of the long version	77.8 ± 24.6	72.1 ± 25.4	<0.001	0.30
Total score of the short version	26.4 ± 8.4	23.6 ± 8.7	<0.001	0.33

Values represent the mean ± standard deviation. The ERD experienced by patients was assessed using the QERD-P, which contains 21 items across 7 factors, with 3 items for each factor, rated on a 7-point Likert scale. High scores indicate worse distress. Boldfaced items indicate those belonging to the short version of the questionnaire. ERD, eating-related distress; QERD-P, Questionnaire for Eating-Related Distress among Patients with advanced cancer.

**Table 4:** Comparison of the QOL before and after palliative care team interventions (*n* = 122).

	Baseline	Week 1	*p*-Value	Effect Size
Functional scales				
EORTC QLQ-C30, Physical functioning	52.3 ± 26.3	52.3 ± 29.4	0.532	0.03
EORTC QLQ-C30, Role functioning	34.0 ± 30.5	41.8 ± 33.8	0.036	0.23
EORTC QLQ-C30, Emotional functioning	61.8 ± 23.4	64.4 ± 25.7	0.189	0.11
EORTC QLQ-C30, Cognitive functioning	54.8 ± 26.8	57.7 ± 30.5	0.236	0.12
EORTC QLQ-C30, Social functioning	52.9 ± 32.0	52.4 ± 32.1	0.722	0.04
Symptom scales				
EORTC QLQ-C30, Fatigue	69.9 ± 22.7	62.4 ± 24.9	0.001	0.31
EORTC QLQ-C30, Nausea and vomiting	22.7 ± 27.2	20.7 ± 27.3	0.395	0.08
EORTC QLQ-C30, Pain	70.7 ± 30.4	56.8 ± 29.9	<0.001	0.45
EORTC QLQ-C30, Dyspnea	46.6 ± 35.4	42.1 ± 32.4	0.065	0.11
EORTC QLQ-C30, Sleep disturbance	59.8 ± 35.2	51.8 ± 34.1	0.027	0.25
EORTC QLQ-C30, Appetite loss	65.8 ± 33.5	57.8 ± 31.7	0.023	0.25
EORTC QLQ-C30, Constipation	43.7 ± 34.1	45.0 ± 38.1	0.908	0.02
EORTC QLQ-C30, Diarrhea	25.3 ± 31.5	21.1 ± 28.6	0.151	0.14
EORTC QLQ-C30, Global health status	28.4 ± 21.7	38.9 ± 23.4	<0.001	0.50
EORTC QLQ-C30, Financial difficulties	40.0 ± 36.3	38.1 ± 37.4	0.718	0.06

Values represent the mean ± standard deviation. QOL was assessed using the EORTC QLQ-C30. High scores indicate a higher quality of life in terms of the functional scales and global health status, or worse symptoms and difficulties in terms of the symptom scales and financial difficulties. QOL, quality of life; EORTC QLQ-C30, European Organization for Research and Treatment of Cancer Core Quality of Life Questionnaire.

**Table 5:** Comparison of the effects of palliative care team interventions between the High- and Low-NIS groups.

	Number of NISs at Baseline			
	Low-NIS Group (*n* = 35)	High-NIS Group (*n* = 88)	*p*-Value	Effect Size
Change in the number of NISs with a score ≥ 4	2.3 ± 3.0	−1.1 ± 3.6	<0.001	0.99
Improvement rate of each NIS *				
Oral pain	1 (2.9)	3 (3.4)	0.856	0.02
Pain	5 (14.3)	33 (37.5)	0.021	0.21
Shortness of breath	1 (2.9)	17 (19.3)	0.023	0.21
Fatigue	1 (2.9)	30 (34.1)	<0.001	0.34
Drowsiness	1 (2.9)	23 (26.1)	0.006	0.25
Lack of appetite	4 (11.4)	35 (39.8)	0.002	0.28
Early satiety	5 (14.3)	27 (30.7)	0.058	0.17
Nausea	0 (0.0)	19 (21.6)	0.003	0.27
Vomiting	0 (0.0)	11 (12.5)	0.032	0.20
Constipation	1 (2.9)	18 (20.5)	0.015	0.22
Diarrhea	1 (2.9)	12 (13.6)	0.084	0.16
Abnormal taste	0 (0.0)	16 (18.2)	0.006	0.25
Abnormal smell	0 (0.0)	7 (8.0)	0.088	0.15
Dry mouth	0 (0.0)	18 (20.5)	0.004	0.26
Dental problems	1 (2.9)	10 (11.4)	0.136	0.13
Difficulty swallowing	2 (5.7)	6 (6.8)	0.777	0.03
Food bolus obstruction	0 (0.0)	12 (13.6)	0.025	0.20
Anxiety	1 (2.9)	26 (29.5)	0.002	0.29
Sadness	0 (0.0)	32 (36.4)	<0.001	0.37
Number of improved NISs	1.3 ± 1.3	5.5 ± 3.4	<0.001	1.42
Change in dietary intake scores	−0.2 ± 2.5	0.5 ± 2.4	0.100	0.30
Improvement rate of dietary intake 1 **	4 (11.4)	10 (11.4)	0.950	0.01
Improvement rate of dietary intake 2 ***	9 (25.7)	24 (27.3)	0.929	0.01
Change in QERD-P scores	−5.7 ± 20.3	−8.4 ± 19.3	0.617	0.26

Values represent *n* (%) or the mean ± standard deviation. Patients having four or more NISs with a score ≥ 4 and those having less than four NISs with a score ≥ 4 were categorized into the High- and Low-NIS groups based on their baseline scores. * The percentage of individuals who improved to a symptom score < 7 among those who had a score ≥ 7 at baseline, and individuals who improved to a score < 4 among those who had a score ≥ 4 at baseline. ** The percentage of individuals whose dietary intake score changed from ≤7 to >7. *** The percentage of individuals whose dietary intake score increased by 2 points. A decrease in the number of NISs with a score ≥ 4, an increase in the improvement rate, and an increase in the change in dietary intake scores indicate an improvement. NIS, nutrition impact symptom; QERD-P, Questionnaire for Eating-Related Distress among Patients with advanced cancer.

**Table 6:** Multiple regression analyses for the improvement in dietary intake and ERD (*n* = 123).

Dietary intake				
Independent variables	B	β	95% CI	*p*-value
Constant	0.19		−3.44, 3.82	0.918
Number of NISs with a score ≥ 4 at baseline (0–3 vs. 4 or more)	1.08	0.19	0.03, 2.14	0.044
Age	−0.02	−0.09	−0.05, 0.02	0.359
Sex (male vs. female)	0.38	0.07	−0.64, 1.39	0.464
Primary cancer site (lung, gastrointestinal tract vs. other)	−0.41	−0.14	−0.99, 0.16	0.158
ECOG performance status (0/1, 2 vs. 3/4)	−0.04	−0.01	−0.66, 0.58	0.901
Treatment status (pre-chemotherapy, chemotherapy vs. never treated/previous treatment)	−0.20	−0.04	−1.15, 0.74	0.669
ERD				
Independent variables	B	β	95% CI	*p*-value
Constant	−1.71		−29.06, 25.65	0.902
Number of NISs with a score ≥ 4 at baseline (0–3 vs. 4 or more)	−3.42	−0.08	−11.39, 4.55	0.397
Age	−0.01	−0.01	−0.27, 0.25	0.921
Sex (male vs. female)	1.38	0.04	−6.29, 9.05	0.722
Primary cancer site (lung, gastrointestinal tract vs. other)	1.56	0.07	−2.84, 5.97	0.483
ECOG performance status (0/1, 2 vs. 3/4)	1.30	0.05	−3.42, 6.02	0.587
Treatment status (pre-chemotherapy, chemotherapy vs. never treated/previous treatment)	−3.49	−0.10	−10.63, 3.64	0.334

Dietary intake was assessed using the IVVAS (10-point scale). High scores indicate better dietary intake. The ERD experienced by patients was assessed using the QERD-P. High scores indicate worse distress. The number of NISs with a score ≥ 4, age, sex, primary cancer site, ECOG performance status, and treatment status were included in the multivariate analysis. ERD, eating-related distress; NIS, nutrition impact symptom; IVVAS, Ingesta-Verbal/Visual Analogue Scale; ERD, eating-related distress; QERD-P, Questionnaire for Eating-Related Distress among Patients with advanced cancer; ECOG, Eastern Cooperative Oncology Group; B, partial regression coefficient; β, standardized partial regression coefficient; CI, confidence interval.

## Data Availability

The original contributions presented in this study are included in the article. Further inquiries can be directed to the corresponding author.
